# Fabrication of Silver Nanoparticles Using a Gas Phase Nanocluster Device and Preliminary Biological Uses

**DOI:** 10.3390/ma11122574

**Published:** 2018-12-18

**Authors:** M. Mery, N. Orellana, C. A. Acevedo, S. Oyarzún, F. Araneda, G. Herrera, D. Aliaga, W. Creixell, T. P. Corrales, C. P. Romero

**Affiliations:** 1 Centro Científico Tecnológico de Valparaíso-CCTVal, Universidad Técnica Federico Santa María, Av. España 1680, Valparaíso 2340000, Chile; mf.meryduarte@gmail.com (M.M.); cristian.acevedo@usm.cl (C.A.A.); david.aliaga@usm.cl (D.A.); werner.creixell@usm.cl (W.C.); 2 Departamento de Física, Universidad Técnica Federico Santa María, Av. España 1680, Valparaíso 2340000, Chile; tomas.corrales@usm.cl; 3 Departamento de Física, Universidad de Santiago de Chile (USACH), Santiago 9170124, Chile; simon.oyarzun@usach.cl; 4 Center for the Development of Nanoscience and Nanotechnology, CEDENNA, Estación Central, Santiago 9170124, Chile; fabian.araneda@usach.cl; 5 Centro de Biotecnología “Dr. Daniel Alkalay Lowitt”, Universidad Técnica Federico Santa María, Av. España 1680, Valparaíso 2340000, Chile; nicole.orellana@usm.cl; 6 Departamento de Física, Facultad de Ciencias Físicas y Matemáticas, Universidad de Chile, Blanco Encalada 2008, Casilla 487-3, Santiago 8370449, Chile; guillermo.herrera.huerta@gmail.com

**Keywords:** metallic clusters, gas phase clusters, nanoparticles, silver clusters, myoblast cells

## Abstract

Nanoparticles can be used in a large variety of applications, including magnetic sensing, biological, superconductivity, tissue engineering, and other fields. In this study, we explore the fabrication of gas phase silver nanoparticles using a sputtering evaporation source. This setup composed of a dual magnetron cluster source holds several advantages over other techniques. The system has independent control over the cluster concentration and a wide range of cluster size and materials that can be used for the clusters and for the matrix where it can be embedded. Characterization of these silver nanoparticles was done using transmission electron microscopy (TEM). We obtain a lateral width of 10.6 nm with a dispersion of 0.24 nm. With atomic force microscopy (AFM) a Gaussian fit of this distribution yields and average height of 6.3 nm with a standard deviation of 1.4 nm. We confirm that the deposited silver nanoparticles have a homogenous area distribution, that they have a defined shape and size distribution, and that they are single standing nanoparticles. Given that the scientific literature is not precise regarding the toxic concentration of the nanoparticles, devices such as ours can help clarify these questions. In order to explore further biological applications, we have done preliminary experiments of cell spreading (myoblast adhesion), obtaining interesting morphological changes correlated with the silver concentration on the surface. With a deposited silver concentration ranging from 100–620 ng/cm^2^, the cells showed morphological changes in a short time of 2 h. We conclude that this high precision nanoparticle fabrication technique is adequate for further biological research.

## 1. Introduction

Metallic Nanoparticles (NPs) have relevance in a varied range of areas, such as developing catalytic converters and solar cells [1]. The production of such metallic NPs in the gas phase has been studied for several years and is very relevant today. Presently, many types of gas phase cluster sources are available [2] and deposited cluster properties, ranging from magnetism, superconductivity, and optical properties to catalysis, are being investigated. In this work we have used one such method to produce metallic clusters-assembled nanostructures in a new setup installed at the Physics department of Universidad Técnica Federico Santa María (USM), Valparaiso Chile. This is the first equipment with this feature in Chile and with its size selective capabilities is probably a first in South America. This design is based on existing devices and was fabricated by Sinoraybo NanoTech of China (Suzhou, Jiangsu, China) [3].

There are many ways of possessing NPs today, a whole array of traditional methods exist: chemical etching (an acidic solution is applied which then eats away at the metal, leaving the etched pattern or design remaining), sputter gas phase (explained in this work), laser ablation (evaporation of material is done through the use of Laser pulses), etc. Several texts regarding these methods are available. For further in-depth description please refer to references [4,5]. All these methods have positive aspects as well as negative ones. We use the gas phase sputter device installed at USM due to its very precise control of deposited material.

Using NPs in combination with biological elements is not a new thing. It has previously been utilized, for example, to understand the fluorescent property and biocompatibility of fluorescent gold nanoclusters in human aortic endothelial cells [6], or to have NPs that can metabolize radiosensitizers for cancer radiotherapy [7]. The development of products based on NPs for medical applications has been an emerging field. Many kinds of NPs have been used. For example, composites made of bioactive ceramic, such as hydroxyapatite (HA) and titanium dioxide (TiO_2_), have been used in bone regeneration [8]. Metallic NPs such as silver (Ag) have been blended with scaffolds for skin repair and to prevent bacterial infections in vitro [9] and in vivo [10]. Another important characteristic of the use of NPs is their cytotoxicity. The scientific literature is not very precise about the toxic concentration of the NPs [9]. However, the cytotoxicity is different if the nanoparticles have been dissolved in the cell culture medium or if they are linked to a surface. The current widespread exposure to NPs has prompted diverse sectors of society to raise questions about what the safe level of NPs actually is for humans and the environment [11,12,13].

In the case of silver NPs, the interest resides as an antimicrobial agent in everyday use products such as refrigerators and washing machines. In the medical sector, they are used for wound dressings and coatings in medical devices. Today it is even possible to buy t-shirts with nanosilver [14]. The most pronounced effect of silver nanoparticles and the role of particle size are still being investigated today. Due to the complexity of the interactions in living organisms these effects are still far from being understood. M. Park et al. [15] studied the effects of silver nanoparticles of different sizes (20, 80, 113 nm) and compared in in vitro assays for cytotoxicity, inflammation, genotoxicity, and developmental toxicity. The results showed that “effects of silver nanoparticles on different toxic endpoints may be the consequence of their ability to inflict cell damage. In addition, the potency of silver in the form of nanoparticles to induce cell damage compared to silver ions is cell type and size-dependent” [15]. What is needed to close this knowledge gap, is to develop toxicity experiments with individual types of cells and silver NPs.

Our group has, in previous work, used NPs solely for solid-state physics topics, such as magnetism, superconductivity, and hydrogen storage [16,17,18]. This incursion into this specific topic of mixing biological response and NPs is due to the relevance we see in this unfolding field.

In this paper, we have combined the gas phase nanocluster production with a specific study of cell spreading. Biological interaction of silver (Ag) NPs was studied by means of the myoblast adhesion. Several samples were made with different concentrations of NPs. The trends of this behavior were observed and are reported in this work. First, we provide evidence of the production of metallic clusters on diverse substrates, then use Ag clusters to carry out a biocompatibility test using cell spreading.

## 2. Materials and Methods

### 2.1. Cluster Equipment, Working Principles of the Nanoparticle System for Sample Production

Cluster-assembled nanostructures are prepared in a gas phase cluster source. The experimental process is described briefly as follows. This source operates on the principle of quenching of a hot metal vapor in a flowing stream of cool inert gas. Metal atoms condense, producing clusters with a broad distribution of sizes and low internal temperature. Clusters are then extracted into a high vacuum region through a nozzle at the end of an aggregation chamber. The gas condensation cluster source is capable of producing clusters containing anything from 2 to 10^5^ atoms each. Thus, this type of source is among the most flexible in terms of cluster sizes. The mass selection of the cluster beam is performed by a high transmission time of flight mass filter, which can resolve close to 2 amu [19,20].

**Figure 1:**
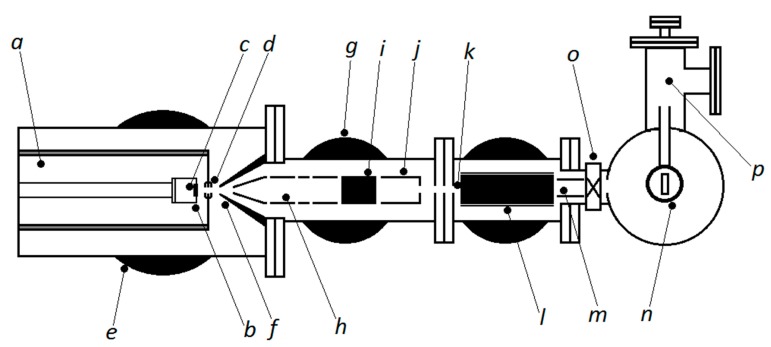
Scheme of the cluster source, showing (**a**) condensation chamber; (**b**) magnetron sputtering head; (**c**) metal target; (**d**) nozzle; (**e**) turbo pump; (**f**) skimmer; (**g**) turbo pump; (**h**) acceleration and focus lens, (**i**) X-Y deflector; (**j**) second focus lens; (**k**) entrance of the mass filter; (**l**) high transmission mass spectrometer; (**m**) set of einzel lens; (**n**) holder sample; (**o**) gate valve; (**p**) transfer chamber.

In Figure 1, we show a scheme of the cluster source. The cooling inert gas (He, N, Ar, or a mix of them) enters the aggregation chamber (a) through three independent gas lines. Inside the chamber is the magnetron sputtering dual head (b) and the sputtering metal targets (c), these are surrounded by a gas entrance. The metal vapor from the sputtered target is cooled by the inert gas and condenses to form clusters, which are swept out through the nozzle (d). The inert gas and vapor mixture undergo a supersonic expansion into a region maintained at a pressure of around 10^−4^ mbar by a turbo molecular pump (e) with a pumping speed of 3000 s^−1^. The ionized beam leaves the condensation chamber and passes through a skimmer (f) to the high vacuum region, pumped by a turbo molecular pump of 2000 s^−1^. This pump is fitted with an integral water-cooled baffle in order to reduce backstreaming of pump oil into the chamber. The beam is accelerated and focused (h), and then aligned with an X-Y deflector (i). A second lens (j) focuses the beam at the entrance of the mass filter (k), to produce a minimum spot size, which improve the mass resolution. The mass of the cluster is selected by a high transmission mass spectrometer (l) which disperses the beam in the vertical plane according to the mass to charge ratio. The mass-selected clusters are separated from the neutral clusters by electrically deflecting them. A set of einzel lenses (m) are used to steer the beam onto the center of the sample holder (n). A gate valve (o) together with a transfer chamber (p) allows for a rapid sample exchange without the need to vent the cluster source.

In the condensation chamber, the metal–gas mixture coalesces to form the metal clusters. The magnetron sputtering dual head permits the evaporation of two different targets at the same time, thus enabling the synthesis of bi-metallic clusters. The most significant parameters to determine the cluster formation are: the inert gas pressure and temperature, the inert gas flow rate, the distance from the magnetron sputtering head to the nozzle, and the sputtering power. Adjusting these parameters determines the most probable size and the efficiency of cluster production. 

### 2.2. Sample Fabrication: Ag Nanoparticles

Synthesis of Ag was performed using the condensation cluster source previously described; they were fabricated in different runs of the cluster setup. Ag NPs were deposited onto different substrates, e.g., lacey carbon grids for Transmission Electron Microscopy (TEM) and SiO_2_ for AFM measurements. The inert gas used for cooling the metallic vapor is Ar at a pressure of 0.1 mbar and at room temperature 22 °C. This mixture of gas and metal atoms is produced in the condensation chamber. The sputtering head and the nozzle are separated by 13 cm and the sputtering power used was 20 W. Once the condensed nanoclusters leave the nozzle, a beam of the particles is formed. With these parameters we obtain a maximum current of ion cluster that is aligned, focused, and guided to the sample holder. The time of deposition ranges from a few seconds up to 3000 s.

Five kinds of samples were fabricated. Ag clusters with a mean size of 9.0 nm ± 2.8 nm were deposited during 5 different exposure times shown in Table 1. During fabrication of these 5 samples, two issues were addressed. Firstly, to deposit NPs on the substrates, and secondly to attach to each sample to a TEM grid. This grid allows the estimation of the size of the clusters and the amount of mass deposited. With the size distribution and the number of clusters per area, one can estimate the equivalent mass of Ag deposited on each sample, these results are expressed in nanograms per cm^2^. Results are shown in Table 1 (see in Supplementary Materials, Figure S1).

### 2.3. Sample Characterization

Atomic force microscopy (AFM) measurements were performed both in static and dynamic modes. Contact mode AFM measurements were carried out using an Omicron UHV AFM/STM (Scienta Omicron, Taunusstein, Germany) at 0.5 nN operating at room temperature in high vacuum conditions (10^−7^ mbar). Dynamic mode AFM measurements were performed using a Nanowizard 3 AFM (JPK, Berlin, Germany), which was operated in quantitative imaging mode. Dynamic mode images are obtained at a 1 nN force set point using PointProbe® Plus (PPP-CONTSCAuD) cantilevers from Nanosensors (Neuchâtel, Switzerland). AFM images were processed using Gwyddion software (Czech Metrology Institute, Brno, Czech Republic).

All the Transmission Electron Microscopy measurements (TEM) were performed using a Hitachi HT7700 system working at 120 keV (Hitachi, Tokyo, Japan).

### 2.4. Cell Culture

The myoblast cell line C2C12 was used as model of mammal cells. The cell line was purchased from the European Collection of Cell Cultures (ECACC) and supplied by Sigma-Aldrich (St. Louis, MO, USA). Cells were cultured at standard conditions (37 °C and 5% CO_2_ in a humidified atmosphere) using DMEM as cell culture medium (Gibco, Life Technologies, Grand Island, NY, USA), supplemented with 10% fetal bovine serum (Biologicals Industries, Kibbutz Beit-Haemek, Israel) and antibiotics (100 U/mL of penicillin and 100 µg/mL of streptomycin).

### 2.5. Cell Spreading and Morphological Assay

Before the spreading experiment, the cells were serum deprived for 2 h. Then, they were suspended by trypsinization and seeded onto poly-l-lysine coated glass slides (2 × 10^5^ cell/cm^2^) using DMEM (without serum) for 2 h at 37 °C [21,22]. The slides were previously treated with the gas phase methodology to deposit the silver nanoparticles.

The cells were fixed with 4% paraformaldehyde for 20 min and washed three times with washing solution (50 mM Tris buffer pH 7.6 containing 0.15N NaCl and 0.1% sodium azide). Cells were permeabilized with 0.1% Triton X-100 in washing solution for 10 min and washed twice.

Rhodamine–phalloidin 1:100 (Invitrogen, Thermo Fisher Scientific, Eugene, OR, USA) was used to stain polymerized actin. Hoechst 33342 (1:10,000) (Invitrogen, Thermo Fisher Scientific, Eugene, OR, USA) was used for nuclear staining. Slides were washed, mounted with ProLongTM Antifade Mountant (Invitrogen, Thermo Fisher Scientific, Eugene, OR, USA), and visualized with an inverted fluorescence microscope (Nikon, Eclipse TS2FL, Tokyo, Japan). Image analysis of the cells was done by using ImageJ Software (NIH, version 1.51k, Bethesda, MD, USA).

Two general parameters were observed. The first is the cell density on the samples and the second is the cell morphology. These parameters ought to present variations when compared to samples without Ag NPs. At least 100 cells were measured for each kind of sample. This was done on 5 different viewing fields on each sample. Results for each sample are shown as the average of these measurements.

### 2.6. Statistical Analysis

The experimental data were fitted using a linear regression. The curve obtained was plotted considering a confidence band of 95%. The significance of the regression (*p*-value) was informed together with the determinant coefficient (R2).

## 3. Results and Discussion

### 3.1. Nanoparticle Sample Fabrication

**Figure 2:**
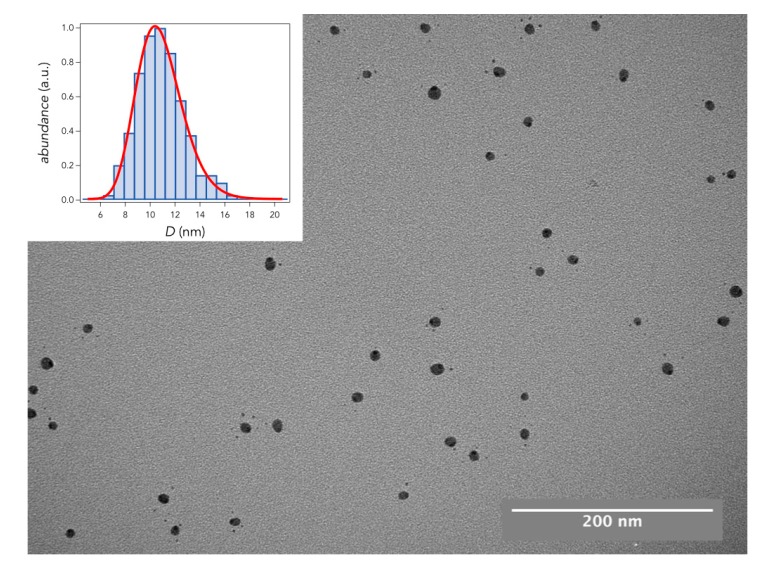
TEM image of Ag clusters deposited on a lacey carbon transmission grid. The inset shows the size distribution of the deposited NPs.

On all our samples we have performed both TEM and AFM measurements. In Figure 2, we show a representative TEM image of an Ag cluster sample prepared with a deposition time of 100 s. The inset of Figure 2 shows a histogram with the lateral diameter of these NPs as measured by TEM. We obtain a lateral width of 10.6 nm with a dispersion of 0.24 nm. 

**Figure 3:**
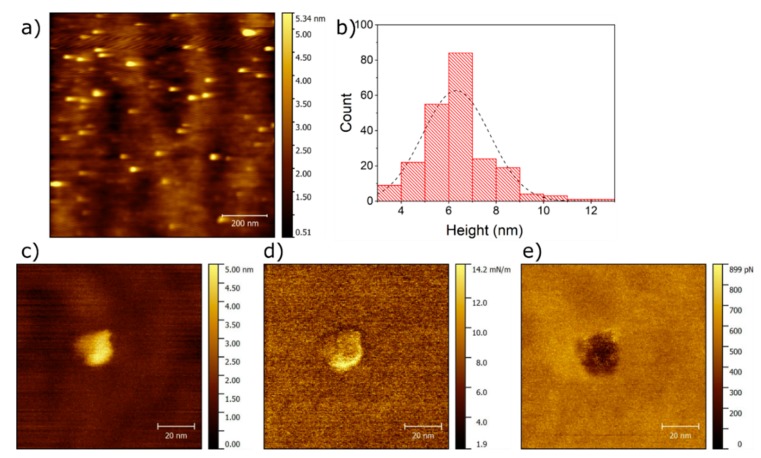
AFM image of Ag clusters deposit on a SiO_2_ wafer substrate. (**a**) Topographical height obtained in contact mode; (**b**) Histogram of Ag NPs from contact-mode images; (**c**) Height in QI^TM^ mode; (**d**) Elastic constant in QI mode; (**e**) Adhesion in QI mode.

Additionally, AFM measurements on Ag NPs deposited on SiO_2_ were performed. Contact mode images are shown in Figure 3a. By masking images taken in contact mode and extracting the maximum height of the marked grains, a total of 222 NPs were measured in height. Figure 3b shows a histogram of height distribution of Ag NPs. A Gaussian fit of this distribution yields and average height of 6.3 nm with a standard deviation of 1.4 nm. Single Ag NPs are measured in QI^TM^ mode. Using this AFM operation mode, we can obtain the height (Figure 3c), elasticity (Figure 3d), and adhesion (Figure 3e). The height of this single Ag NPs is roughly 5 nm, while its elastic constant is slightly higher than the SiO_2_ background. Furthermore, the adhesion of the Ag NPs is lower than the SiO_2_ background. From Figure 3d,e we confirm that the imaged NPs are composed of a different material than the background.

From the TEM and AFM images we conclude that our Ag NPs are on average 10 nm in width and 6 nm in height. This means that our clusters are not perfectly spherical. In the following sections we shall study their biological response to our Ag NPs.

### 3.2. Biological Response of Myoblast Cells

**Figure 4:**
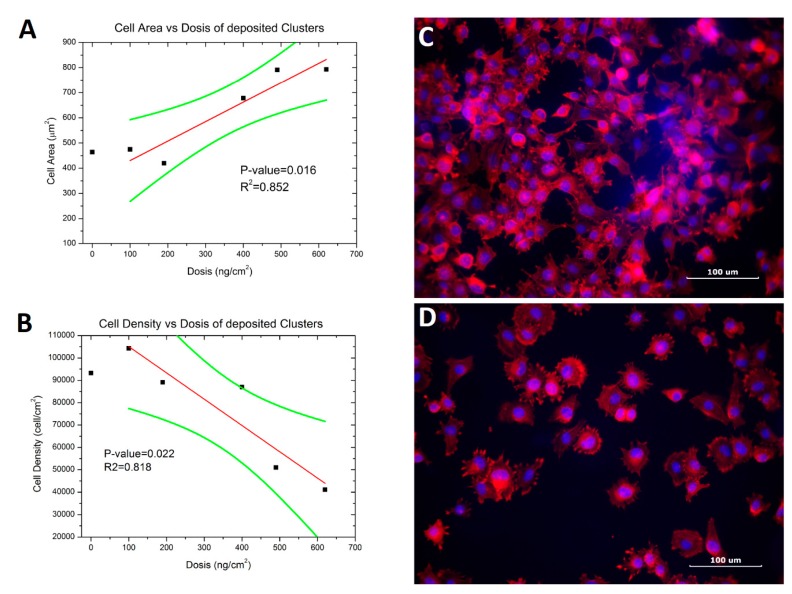
Cell spreading test on glass with nanoclusters of Ag. (**A**) Shows the cellular adhesion density on the different samples as a function of Ag concentration. The red line is the linear fitting and the green is the confidence band (95%). (**B**) Shows the adhered cell size as a function of Ag concentration. The red line is the linear fitting and the green is the confidence band (95%). (**C**) Fluorescence image of cells on the 100 ng/cm^2^ sample 5. (**D**) Fluorescence image of cells on the 620 ng/cm^2^ sample 1. In all images, the linear fit of the blank sample was not included.

The biological response of silver NPs was studied by means a simple cell spreading experiment. Morphological observation and cell counting were done to evaluate the effect of silver nanoparticle deposition. Myoblast cells in suspension were allowed to attach onto the glass cover with the deposited silver NPs. Results are displayed in Figure 4, showing strong dependence between cluster concentration and cell behavior.

In the cell spreading after the trypsinization, three steps are distinguished: (a) blebbing of rounded cells or first attachment to a solid substratum; (b) organization of a peripheral zone of actin fibrils; and (c) extension of lamellae [23].

Shown in Figure 4A, we display the cellular adhesion density on the different samples as a function of Ag concentration after 2 h of exposition to the substrates. A negative correlation is obtained, suggesting a mild tendency of toxicity on the cells due to the presence of Ag clusters on the samples. In Figure 4B we display the adhered cell size as a function of Ag concentration, also after 2 h of exposition to the substrates. It is observed that there exists a positive correlation, and in this case, there is a strong effect on the cell morphology due to the presence of the Ag on the substrates. In Figure 4C, we show a fluorescence image of adhered cells on the 100 ng/cm^2^ substrate (sample 5). It can be seen that there is a high cellular density with a star-like and fibroblastic morphology typical of the cell line (Burattini et al. 2004) [24]. The blue color corresponds to nuclear staining and the red to actin staining. In Figure 4D, we show a fluorescence image of adhered cells on the 620 ng/cm^2^ (sample 1). Here, it is evident that there is a low cellular density with a spherical morphology. This suggests that the presence of the Ag clusters at higher densities does affect the morphology that the cells exhibit. It has been reported that morphology of C2C12 cells depend of the kind of surface culture, e.g. fibroblastic shape on plastic and rounded on Matrigel [25]. In the Table 1 (see in supplementary material we present images of the cell adhesion of samples without NPs and Samples 2, 3, and 4. Samples 1 and 5 are shown in Figure 4C,D.

It is well known that larger Ag NPs (20–80 nm) release Ag ions generating toxic effects. Smaller NPs also release Ag ions but show greater toxicity than larger NPs [26]. Further, the cytotoxicity levels are not clear. In reference [11], the IC50 concentration has been determined to be 55 μg/mL and 1408 μg/mL, depending on what laboratory was used for examination. This is an important distinction because the current literature is regarding concentration in volume (3-D), whereas our approach is mass on a surface (2-D). 

The phenomenon of adhesion has also been reported by other authors. They claimed that Ag NPs impregnated on poly(l-lactide) were anti-adhesive on fibrous membranes [27].

In our experiment, most of the NPs are smaller than 12 nm. The results show that our Ag NPs have moderate biological effects, even when increasing the amount of deposited Ag by more than 6-fold, from 100 to 620 (ng/cm^2^).

## 4. Conclusions

The newly created nanocluster laboratory at USM can produce metallic NPs. We show evidence of Ag NPs with mean sizes of 9.0 nm ± 2.8 nm. The Ag NPs were deposited onto sterile glass covers, TEM grids, and SiO_2_. The glass samples were exposed to mammalian cells for 2 h and cell spreading was studied. It was shown that when the concentration of deposited Ag NPs is 620 ng/cm^2^, there is a significant change in the density of cells on the samples as well as a morphologic effect. The cells change from their typically expanded morphology to a spherical one. There is also a lower number of cells per unit area. This lower density is not as significant as the morphological changes. The reason for this may be attributed to the fact that our Ag clusters do not spread out as atomic Ag would, thus creating less contact points for each cell with an Ag atom. The literature reports that Ag NPs smaller than 10 nm have very high toxicity, although this is not clear in our experiments, even when we increased the amount of Ag NPs by 6 times. We remind the reader that a significant part of our NPs that were deposited are below 10 nm. 

Future experiments will involve the creation of a mix of Cu-Ag NPs to establish if these present a better tool for bactericidal surfaces. The experimental setup at USM, Valparaiso enables the manufacturing of NP alloys due to its dual-sputter head. This is a unique feature of gas phase cluster setups. Further experiments using silver NPs will also be prepared but with the mass-selecting ability, thus creating samples with different exposures of NPs and different mean sizes. 

## Figures and Tables

**Table 1:** Sample fabrication parameters.

Sample	Deposition Time (s)	Observed Average Coverage (ng/cm^2^)
1	2389	620
2	2160	400
3	1237	490
4	750	190
5	316	100

## Supplementary Materials

The following are available online at http://www.mdpi.com/1996-1944/11/12/2574/s1, Figure S1: additional fluorescence image of cell cultures.
