# Supplementary material for: Fabrication of Silver Nanoparticles Using a Gas Phase Nanocluster Device and Preliminary Biological Uses
Source: Materials (Basel). 2018 Dec 18;11(12):2574. doi: 10.3390/ma11122574 (PMC6316590; doi:10.3390/ma11122574)

Supplementary Material

Supplementary material shows additional fluorescence image of cell cultures reported in the manuscript. These are the samples mentioned in Table 1 and the blank sample (Sample without silver NPs).

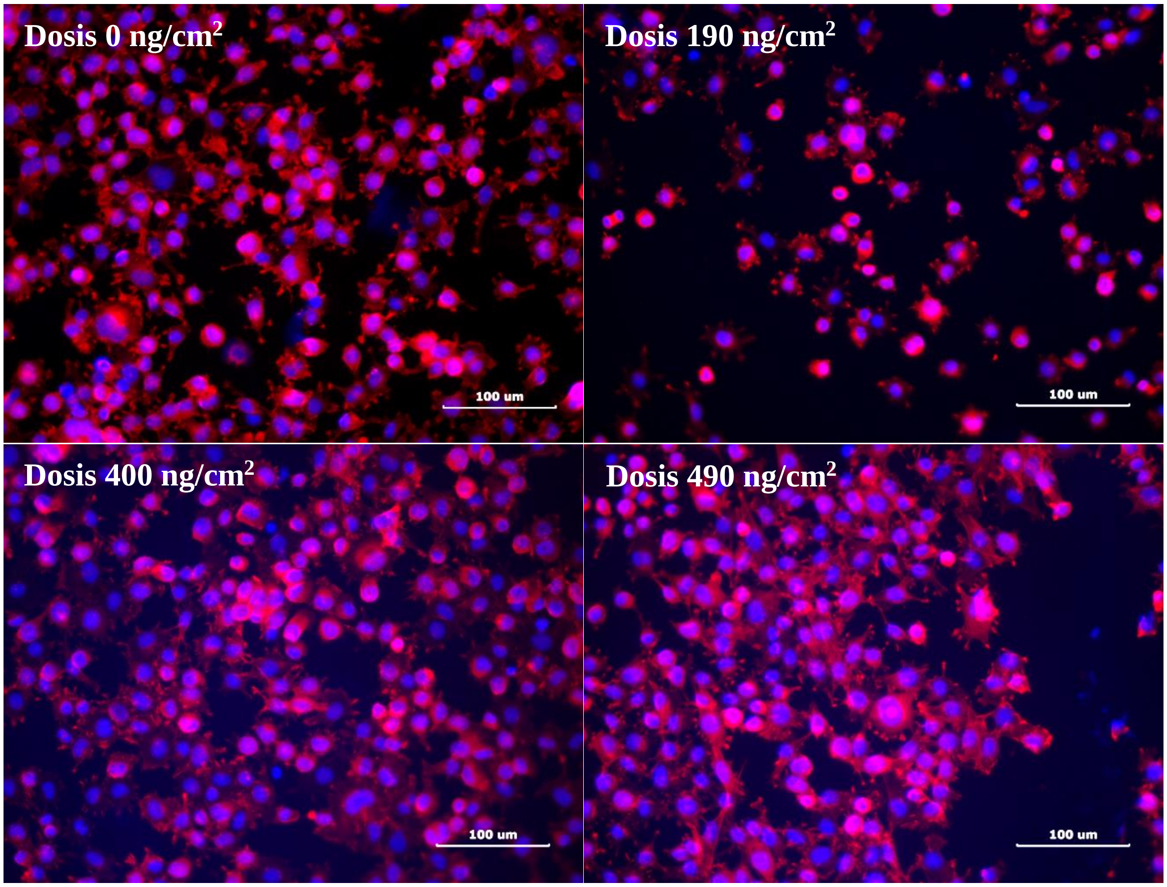

Supplement: Supplementary file 1 [file materials-11-02574-s001.pdf]
